# Aberrant stalk development and breakdown of tip dominance in *Dictyostelium *cell lines with RNAi-silenced expression of calcineurin B

**DOI:** 10.1186/1471-213X-6-12

**Published:** 2006-03-02

**Authors:** Katrina Boeckeler, Gilbert Tischendorf, Rupert Mutzel, Barbara Weissenmayer

**Affiliations:** 1 Institut für Biologie – Mikrobiologie, Fachbereich Biologie, Chemie, Pharmazie, Freie Universität Berlin, Königin-Luise-Strasse 12-16, 14195 Berlin, Germany; 2 University College London, Department of Biology, Gower Street, London, Wc1 E6BT, UK

## Abstract

**Background:**

Calcineurin, the Ca^2+^/calmodulin-dependent protein phosphatase, plays important roles in various cellular processes in lower and higher eukaryotes. Here we analyze the role of calcineurin in the development of *Dictyostelium discoideum *by RNAi-mediated manipulation of its expression.

**Results:**

The *cnbA *gene of *Dictyostelium discoideum *which encodes the regulatory B subunit (CNB) of calcineurin was silenced by RNAi. We found a variety of silencing levels of CNB in different recombinant cell lines. Reduction of CNB expression in a given cell line was correlated with developmental aberrations. Cell lines with strongly reduced protein levels developed slower than wild type cells and formed short stalks and spore heads with additional tips. Formation of short stalks results from incomplete vacuolization of prestalk cells during terminal differentiation. Expression of the stalk-specific gene *ecmB *was reduced in mutant cells. Aberrant stalk development is a cell autonomous defect, whereas the breakdown of tip dominance can be prevented by the presence of as low as 10% wild type cells in chimeras.

**Conclusion:**

Silencing of calcineurin B in *Dictyostelium *by expression of RNAi reveals an unexpected link between increased intracellular calcium levels, possibly triggered by the morphogen DIF, activation of calcineurin, and the terminal stage of morphogenesis.

## Background

Calcineurin (CN), the Ca^2+^/calmodulin-dependent protein phosphatase, is highly conserved from yeast to mammalian cells. It consists of a catalytic (CNA) and a regulatory subunit (CNB) which form a heterodimer upon Ca^2+ ^binding to CNB. The enzyme has been shown to play important roles in various cellular processes in lower and higher eukaryotes. In mammals these include T cell activation via dephosphorylation of the cytosolic component of nuclear factor of activated T cells (NFAT) [1], cardiac development and hypertrophy, learning and memory, and axonal pathfinding [2]. In yeast calcineurin is involved in the regulation of ion homeostasis and cell cycle control [3]. Whereas most organisms have at least two genes for the calcineurin subunits, the *Dictyostelium discoideum *genome contains single copy genes for CNA and CNB, whose expression changes during development of the organism. As in higher organisms CNB is required for high affinity binding of protein substrates by the holoenzyme [4]. CNA is highest in vegetative cells and after aggregation [4,5]. The coding mRNA for CNB is processed by an unorthodox mechanism starting during early development to give rise to a shorter isoform encoding a CNB protein with a truncated N terminus that does not contain the N myristoylation consensus site found in the full-length protein [4]. Several attempts to delete the single gene for CNA (*canA*) in *D. discoideum *by insertion of selectable markers or to reduce its expression by antisense-mRNA failed (U. Kessen and R.M., unpublished results). 30-fold overexpression of the single *cnbA *gene which encodes the regulatory B subunit resulted in moderately accelerated multicellular development with recombinant populations completing morphogenesis about 2–3 hours earlier than wild type cells (F. Fouladi and B.W., unpublished observation). Pharmacological inhibition of *D. discoideum *CN using gossypol impaired growth and cellular signaling [6]. Development of wild type cells treated with 25 μM gossypol was totally blocked for more than 24 hours. Studies using the classical inhibitors, Cyclosporin A (CsA) and FK506, indicated that CN regulates Ca^2+ ^stress-induced transcription activation of the Ca^2+^-ATPase *patA *gene [7] as well as expression of the "calcium upregulated" (Cup) class of Ca^2+^-binding proteins [8]. In an in vitro differentiation assay CsA and FK506 strongly inhibited stalk cell formation in the wild-type and spore formation in a sporogenous *Dictyostelium *strain [9]. These agents also reduced the expression of prestalk and prespore-specific transcripts, assigning a function for CN as a general activator of differentiation

During development *D. discoideum *cells aggregate in response to cAMP, form slugs and differentiate into two major cell types, prespore and prestalk cells. These cells organize in a spatial pattern with the prespore region localizing to the posterior and the organizer, prestalk region to the anterior of the slug. Development is completed by the formation of fruiting bodies consisting of a stalk of dead cells and the spore head. Several lines of evidence link the level of cytosolic Ca^2+ ^(Ca^2+^_i_) to cell type differentiation. Cells in S and early G2 cell cycle phases, which show a tendency to become prestalk cells, have high intracellular calcium [10]. At the slug stage, a gradient of Ca^2+^_i _along the anterior-posterior axis has been found which is inversely correlated with the amount of sequestered Ca^2+ ^and the calcium-sequestering activity in high-capacity Ca^2+ ^stores [11]. High Ca^2+^_i _has been shown to be necessary for induction of the prestalk-specific gene *ecmB *by the morphogen, differentiation-inducing factor (DIF), and a sustained increase in Ca^2+^_i _was proposed to be responsible for prestalk-specific gene expression [12]. This work also showed that the increase in Ca^2+^_i _is brought about by DIF. It is unclear how DIF increases Ca^2+^_i_, and the mechanism by which Ca^2+^_i _ultimately leads to activation of *ecmB *expression has remained elusive.

RNA interference-mediated gene silencing was recently established in *Dictyostelium *[13]. RNAi-mediated silencing of gene expression is induced by double-stranded RNA and its processing to 23 mers which cause the degradation of endogenous target mRNAs [14,15]. The technique appears to be well suited for the analysis of potentially essential genes in the haploid *Dictyostelium *genome since it can lead to different levels of silencing from almost wild type to maximally reduced levels in individual cell lines. This would allow to determine the repression level of a given gene that is just tolerable for cell survival.

We show here that a hairpin RNA construct targeted against *cnbA *effectively reduces the cellular CNB concentration. Recombinant cell lines showed retardation of multicellular development, culminating in fruiting bodies with short stalks and the formation of additional tips on the spore mass of developing fruiting bodies.

## Results

### Isolation of *cnbA *RNAi transgenic cell lines

**Figure 1:**
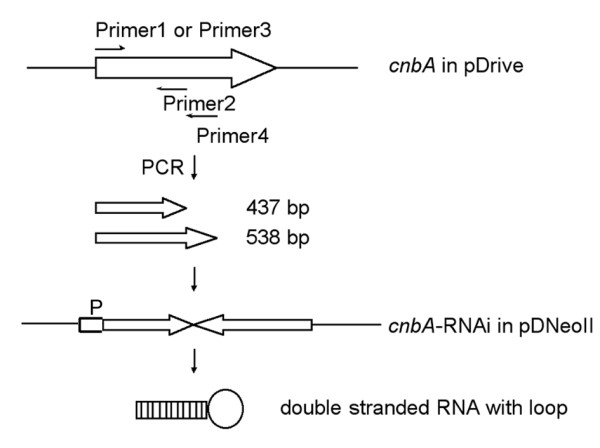
RNAi construct directed against *cnbA*. Fragments of *cnbA *were generated by PCR using oligonucleotide primers flanked by suitable restriction sites. The short and long fragments were amplified with primers 1 and 2 or primers 3 and 4, respectively. They were then fused in opposite orientation and cloned in pDNeoII. See Materials and Methods for further details. P, *actin6 *promotor.

It was previously shown that *cnbA *is a single-copy gene [4] localized on chromosome 1 of *D. discoideum *[16]. In order to silence *cnbA *we constructed a stem-loop RNA directed against *cnbA *(fig. 1). Two fragments of *cnbA *were amplified from the cDNA, both starting at nucleotide +20. To obtain a loop, the fragments differ 100 bp in length. The truncated *cnbA *gene fragments were fused in reverse orientation relative to each other. The RNAi construct was cloned into pDNeoII under the control of the actin 6 promoter. The resulting plasmid pKB07 was transformed in *D. discoideum *AX2 and clonal cell lines were further analysed.

### Downregulation of calcineurin B

**Figure 2:**
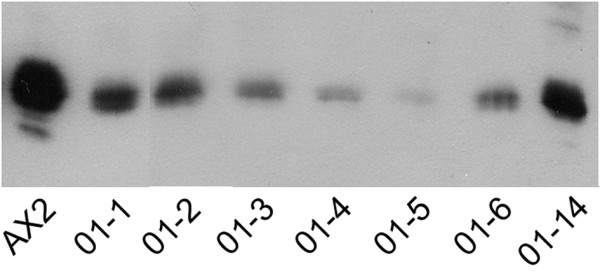
Expression of calcineurin B in wild type cells and *cnbA *RNAi mutants. Total cell protein from 5 × 10^5 ^vegetative cells of the cell lines indicated was separated on 15%polyacrylamide/0.1% SDS gels, blotted onto nitrocellulose, and blots were probed with 1:5000-diluted CNB-specific antibody.

**Figure 3:**
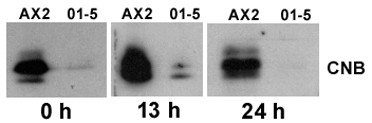
Expression of calcineurin B in wild type and *cnbA *RNAi mutant 01–5 at various time points. Total cell protein was chromatographed on 15% polyacrylamide/0.1% SDS gels, blotted onto nitrocellulose, and blots were probed with 1:5000-diluted CNB-specific antibody.

In order to analyze whether expression of the RNAi construct leads to reduction of CNB, expression of the protein was measured in vegetative cells of independent recombinant clones. Western blots with specific antibodies against *D. discoideum *CNB revealed various levels of reduction among individual cell lines (fig. 2). In a number of cell lines (c.f., 01–4 or 01–5 in fig. 2) CNB was reduced to barely detectable amounts, whereas others (c.f., 0–14 in fig. 2) showed only slightly reduced CNB expression. At later stages during development both CNB protein isoforms were detected (not shown) suggesting that processing of the residual *cnbA *mRNA is not affected by RNAi expression. The expression level of CNB in the mutants remained low throughout development (fig. 3).

### Characterization of *cnbA*-RNAi mutant 01–5

Cell lines with slight reduction of CNB expression displayed no or only mild phenotypic aberrations which were mainly manifest in the erection of fruiting bodies with somewhat shorter stalks than observed with wild type organisms (not shown). However, phenotypic alterations were much more prominent in cell lines with pronounced silencing of CNB. To characterize the phenotypic consequences of silencing of *cnbA *mRNA 10 independent transformants with drastically reduced expression of CNB were further analyzed. The phenotypes of all these transformants were very similar to cell line 01–5 which is described in more detail below.

**Figure 4:**
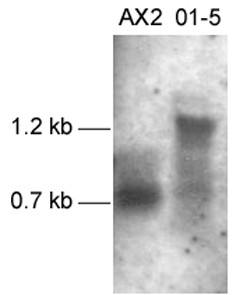
Expression of *cnbA *mRNA in AX2 and 01–5 cells. Total RNA was prepared from cells developed on HABP filters for 8 h, separated on 0.9% agarose/6.6% formaldehyde gels, blotted onto nylon membranes and hybridized with a DIG-labelled probe for *cnbA*. To the left, the sizes of the endogenous processed *cnbA *mRNA species in AX2 (0.7 kb) and of the RNAi product in 01–5 (1.2 kb) are indicated.

We first compared expression of *cnbA *mRNA in cell line 01–5 and parental AX2 cells by Northern blotting. Figure 4 shows that the recombinant cell line had drastically reduced amounts of endogenous *cnbA *mRNA (0.7 kb). A strong signal corresponding to the size of the RNAi construct (1.2 kb) indicates massive expression of the transfected gene. Degradation of both RNAi and endogenous *cnbA *mRNA is indicated by the smear detected in the mRNA isolated from 01–5 cells.

**Figure 5:**
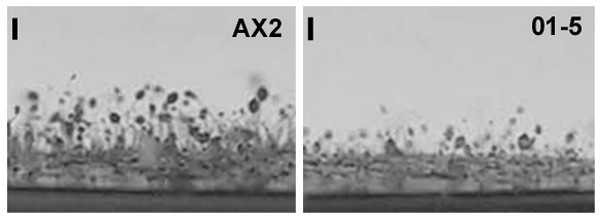
Fruiting body formation in AX2 and *cnbA *RNAi mutant cell line 01–5. Cells were washed twice with SP buffer, 2 × 10^7 ^cells spread onto HABP filters and allowed to complete morphogenesis. A side view of the mature fruiting bodies is shown. Bar, 1 mm.

The growth rate of mutant 01–5 in AX medium was very similar to that of wild type cells, with doubling times of about 9 hours. When these cells developed on non-nutrient agar aggregation was delayed by about 3 hours as compared to wild type cells. The mutant proceeded with normal kinetics through further development, however, 01–5 populations formed longer, leaner slugs than populations of wild type cells (not shown). After termination of development stalks were significantly (ca. 50%) shorter than wild type fruiting bodies (fig. 5). Comparison of mutant and wild type fruiting bodies did not indicate differences in the size of their spore heads. To analyze whether RNAi against *cnbA *indeed affected predominantly stalk size, we compared the numbers of fruiting bodies formed on SP agar in the wells of microtiter plates from 4 × 10^5 ^cells and counted the numbers of spores which could germinate from the mature fruiting bodies. Mutant 01–5 formed 46 ± 20 fruiting bodies (n = 20). From the spores of the fruiting bodies 3.1 × 10^3 ^± 7 × 10^2 ^(n = 5) clones could re-grow on a lawn of bacteria. Wild type AX2 cells formed 61 ± 36 fruiting bodies (n = 20). From the spores of these fruiting bodies 3.1 × 10^3 ^± 10^3 ^(n = 5) clones could re-grow on a lawn of bacteria.

**Figure 6:**
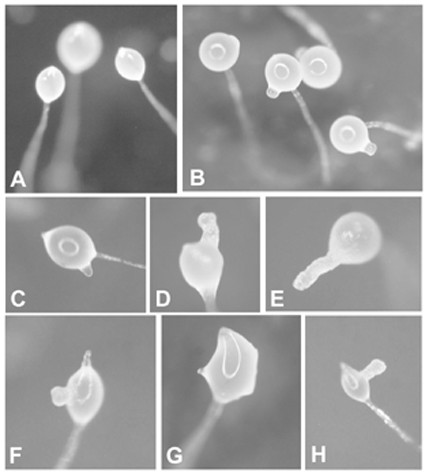
Formation of ectopic tips in culminating fruiting bodies of cell line 01–5. Cells were allowed to develop on SP agar plates and the spore heads photographed using a Nikon SMZ8000 microscope equipped with a Nikon DN100 digital camera. As control AX2 fruiting bodies are shown in A.

In addition to the formation of short stalks, many of the culminants of cell line 01–5 showed one or more additional, irregularly arranged tips protruding from the rising spore heads (fig. 6). These ectopic tips were not observed earlier during development, e.g. on first finger or slug structures but only became visible during culmination. Some of these extra tips (c.f., fig. 6D, E, F, H) are reminiscent of the thicker part of stalks near their base. The tip acts as an organizer region, effectively inhibiting the formation of additional tips [17]. Obviously, dominance of the tip in the RNAi mutant is much weaker than in wild type organisms.

**Figure 7:**
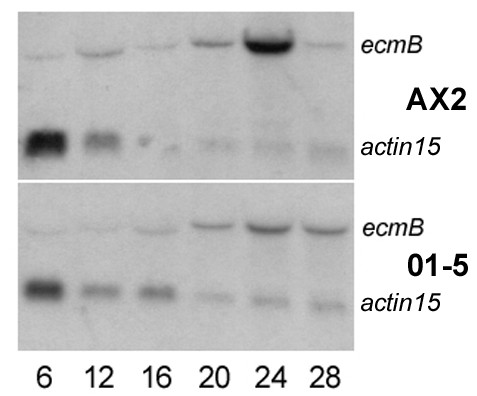
Expression of *ecmB *mRNA in AX2 and 01–5 cells. Total RNA was prepared from cells developed on HABP filters for the times (in hours) indicated, separated on 0.9% agarose/6.6% formaldehyde gels, blotted onto nylon membranes and hybridized with a DIG-labelled probe for *ecmB*. A probe for *actin15 *was used as loading control (signals in the lower part). Note that expression of *actin15 *decreases during development.

Expression of the prestalk-specific gene *ecmB *has previously been shown to depend on an increase in Ca^2+^_i_, making this gene a candidate for transcriptional regulation via CN. Analysis of *ecmB *expression in AX2 and 01–5 cells revealed a significant inhibition of *ecmB *induction during development in the RNAi mutant (fig. 7). In contrast to these findings the expression kinetics of the prestalk-specific marker *ecmA *and of the prespore-specific marker *pspA *were in 01–5 cells similar to wild type cells (data not shown).

### Cell-autonomous and non cell-autonomous effects of RNAi expression

**Figure 8:**
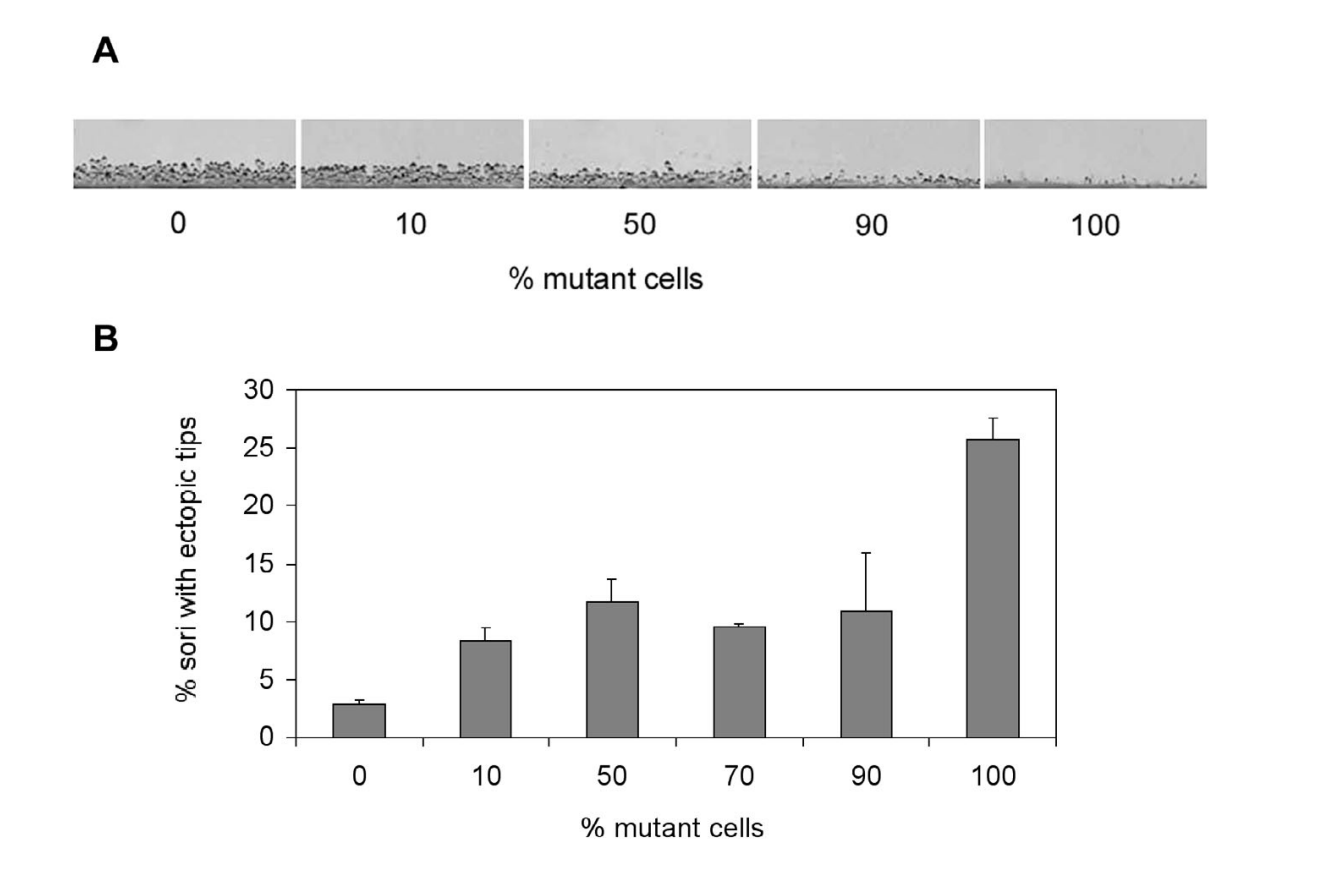
Analysis of chimeras formed from mixtures of AX2 and 01–5 cells. Cell lines were mixed in the ratios indicated and allowed to develop on HABP filters. A. Gradual reduction of stalk size in chimeras containing increasing fractions of cells from line 01–5. A side view of the mature fruiting bodies on HABP filters at low magnification shows that stalk length gradually decreases with increasing fractions of 01–5 cells in chimeras. B. Formation of ectopic tips in chimeras containing increasing fractions of cells from line 01–5. The fractions of fruiting bodies with ectopic tips on an entire HABP filter were counted in a blinded manner. Average values ± SEM from two independent experiments are shown.

To check whether short stalk formation and ectopic formation of extra tips are cell-autonomous or non-autonomous defects, wild type and *cnbA*-RNAi mutant cells were mixed in different ratios and allowed to develop. Stalk size decreased proportionally to increasing content of mutant cells (fig. 8A). This gradual decrease in stalk length suggests that formation of short stalks is a cell-autonomous defect. In contrast, ectopic formation of extra tips on the rising sorophores appears to be at least partially non-cell autonomous (fig. 8B). Only pure mutant populations formed extra tips at high frequencies. Even 10% wild type cells could effectively establish nearly normal tip dominance.

### Analysis of aberrant stalks by light and electron microscopy

**Figure 9:**
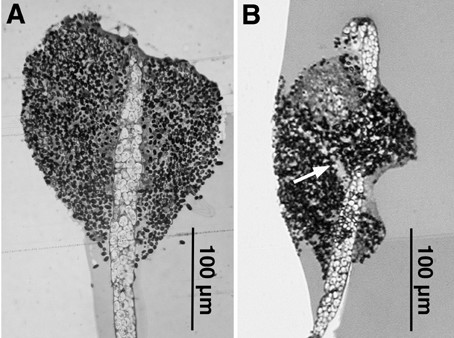
Longitudinal section of wild type (A) and 01–5 mutant (B) fruiting bodies. Spore cells are dark colored, stalk cell bright. The arrow points to an additional stalk-like structure intruding from an ectopic tip.

**Figure 10:**
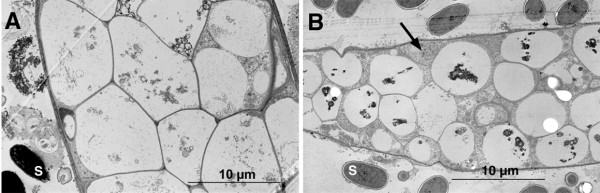
Longitudinal sections of stalks analyzed by electron microscopy. Wild type stalk cells (A, bright cells) are larger than 01–5 mutant stalk cells (B). Note that some of the mutant stalk cells appear to be not vacuolated (arrow). The size of spore cells (S) from wild type and mutant 01–5 (dark or grey cells outside the stalk) are similar.

The examination of sections from fruiting bodies could give information about the origin and composition of the ectopic tips of the *cnbA*-RNAi mutant. Fig. 9 shows a longitudinal section through a wild type fruiting body (A) in comparison to a fruiting body from mutant 01–5 (B). Staining allows a clear discrimination between spore and stalk cells. The dark spore cells are small and condensed, stalk cells are expanded and, because they are highly vacuolated, nearly unstained. In the mutant spore head a channel-structure intruding from the periphery of the spore head can be seen which represents a second stalk arising from an ectopic tip. Since the stalk cells in the mutant seemed to be rounder than wild type stalk cells, electron microscopy examinations were performed. Stalk cells of wild type fruiting bodies (fig. 10A) were clearly larger than mutant stalk cells (fig. 10B). This appears due to incomplete vacuolization of the mutant stalk cells. Some of the mutant stalk cells seemed to be not vacuolated at all. In contrast, the size and form of the spore cells in the wild type and the mutant were closely similar.

## Discussion

The defect in stalk formation which was observed in cell lines with strongly reduced CNB expression occurs due to smaller and incompletely vacuolated stalk cells and this result points to an important role for CNB or the CN holoenzyme in the regulation of stalk development in *Dictyostelium*. DIF-1 induces the expression of prestalk-specific genes via an increase in the intracellular free Ca^2+ ^[12]. Based on our results we speculate that the effects of increased Ca^2+^_i _are mediated by CN. In analogy to the well-characterized role of CN in dephosphorylation of transcription factor complexes of the NFAT type we propose that calcineurin, in response to an increase in cytosolic Ca^2+^, dephosphorylates a cytosolic component of a transcription complex, which is then translocated to the nucleus and activates – in concert with nuclear components of the active complex – the transcription of prestalk genes. This hypothesis is directly supported by our observation that RNAi mutant 01–5 failed to properly induce expression of the prestalk-specific gene *ecmB *during development.

The phenotypic defects which we report here complement previous studies on the effects of mutations in the *Dictyostelium *glycogen synthase kinase-3 homolog GskA [18] which has been shown to promote posterior cell patterning and inhibit anterior cell differentiation [reviewed in [19]]. GskA is regulated through an intracellular protein tyrosine kinase/phosphatase pathway mediated by the cAMP receptors CAR1 and/or CAR3 and CAR4. GskA is activated by CAR1- and/or CAR3-mediated activation of the tyrosine kinase ZAK1 [20], and inactivated by CAR4-mediated activation of a protein tyrosine phosphatase [21]. GskA null cells show accelerated early development and form only stalk cells during morphogenesis [18]. Both the *gskA *null and the *cnbA *RNAi phenotypes are cell autonomous [18]; see above, fig. 8A. The direct link between GskA and CN is supported by recent results that come from analysis of the NFAT pathway. In T cells phosphorylation of NFAT by GSK3 represses NFAT-dependent gene expression by inhibition of NFAT binding to DNA [22]. Inhibition of re-export of NFAT to the cytosol when GSK3 is inactivated, leads to increased NFAT-dependent activation of gene expression [23].

Deletion of the *Dictyostelium aarA *gene, a homolog of mammalian β-catenin, leads to a closely similar breakdown of tip dominance and to formation of ectopic tips during late development as silencing of CNB. In both cases, the defect is non-cell autonomous [24]; see above, fig. 8B. It has been shown that AarA plays a necessary role in the formation of adherence junctions at the neck of the rising stalk [25]. β-catenin is also a transcriptional co-activator in the metazoan canonical Wnt pathway. In the control of dorsoventral axis formation in *Xenopus *by extracellular signals, a CN-independent NFAT mutant inhibited anterior development of the primary axis, whereas a dominant negative NFAT mutation induced ectopic dorsal axis formation and the expression of target genes of the canonical Wnt pathway, suggesting that CN and NFAT are part of the noncanonical Wnt/Ca^2+ ^pathway which leads to inhibition of the canonical Wnt pathway upstream of β-catenin [26] (for a Wnt/Ca^2+ ^pathway overview see [27]). It is therefore possible to propose a model for *Dictyostelium *tip dominance where a pathway similar to the canonical metazoan Wnt pathway is acting through CAR1/CAR3 to activate GskA, and a noncanonical "Wnt/Ca^2+^" pathway through CAR4 to activate CN via an intracellular Ca^2+ ^signal. The postulated transcription factor target for CN in *Dictyostelium *is so far unknown.

Calcineurin B is a homolog of members of the 4 EF hand calcium sensor protein family which include, for example, recoverin, the neuronal calcium sensor-1 (NCS-1), and the plant calcineurin B-like proteins [(CBLs [28]]. It has recently been demonstrated that N myristoylation, a common feature of CNB and the Ca^2+ ^sensor protein family, increases the cooperativity of Ca^2+ ^binding to NCS-1 and leads to larger conformational changes upon Ca^2+ ^binding than in the nonmyristoylated protein [29]. If Ca^2+ ^sensing is indeed a major function of calcineurin B, the phenotypic defects observed in our *cnbA *RNAi mutants may be due to the failure to correctly sense the slow, sustained increase in Ca^2+^_i _from ca. 50 to ca. 150 nM during a period of about 8 hours [12], which was proposed to induce prestalk gene expression. Starting during early development *D. discoideum *cells express two CNB isoforms, one of them lacking the myristoylation consensus site. Further work has to show whether it is the myristoylated CNB isoform which plays a crucial role in the mediation of DIF effects on prestalk gene expression.

## Conclusion

*Dictyostelium *calcineurin B knock down mutants produced smaller stalk cells and as a consequence smaller stalks due to incomplete vacuolization of stalk cells. We propose that calcineurin mediates the effects of a differentiation-inducing factor (DIF)-elicited increase in free intracellular calcium in prestalk cells via the dephosphorylation of a cytosolic transcription complex component which activates the expression of prestalk genes. Culminating fruiting bodies formed ectopic tips on the spore heads which points to a link between calcineurin and the *wnt *pathway in the social amoebae.

## Methods

### Growth and development of *Dictyostelium discoideum*

*D. discoideum *AX2 cells were grown at 22°C in AX medium [30]. Development was induced by washing cells twice with ice-cold Sørensen phosphate (SP) buffer [17 mM (KH_2_/Na_2_H)PO_4_], pH 6.0 and spreading 2 × 10^7 ^cells on HABP nitrocellulose filters (0.45 μm, diameter 47 mm, Millipore, Eschborn, Germany) supported by two paper filters (diameter 47 mm) soaked with buffer.

### Transformation of *Dictyostelium discoideum*

*D. discoideum *AX2 cells were grown in AX medium to a density of 5 × 10^6 ^cells/ml, washed twice with ice-cold H-50 buffer, resuspended at 2 × 10^7 ^cells/ml, and 100 μl of this suspension was electroporated with 10 μg of plasmid DNA [31,32]. Transformed cells were grown on suitable selective media and clonal populations were obtained by serial dilution in microtiter plates. In initial experiments we observed that the phenotypic aberrations which correlated with RNAi expression became gradually weaker upon serial passages of the cells. Therefore, immediately after isolation, clones of recombinant cells were grown to final numbers of approx. 1.5 × 10^9 ^(ca. 31 to 32 generations) and allowed to form spores on SP agar plates which were harvested and kept in SP buffer at -70°C until used to inoculate experimental cultures. These underwent additional 13 to 15 generations.

### Construction of plasmids

*cnbA *cDNA was amplified by reverse transcription of total RNA from vegetative *D. discoideum *using the primer pair 5'GGGCCATGGGGAATCAACATTCATTATTA3' and 5'TTATTCTGACCAATTTACGCTAAG3'. The product was cloned into pDrive (Qiagen, Hilden, Germany) to obtain plasmid pBW104. Construction of the RNAi-encoding plasmid for *cnbA *was done in three steps. First, a fragment of 437 bp amplified with primers 5'CCGGATCCAAGCTTAAAGAACAATTAGAACAAATGA3' and 5'CCGTCGACTCACCTTCAATAATAGTTTTATCAACA3' was cloned in sense orientation into *Bam*HI/*Sal*I-digested pUC18 [33], yielding pKB05. Second, pKB06 was generated by ligating a longer *cnbA *fragment of 538 bp, amplified with primers 5'CCCTGCAGAAAGAACAATTAGAACAAATGA3' and 5'CCGTCGACTTCTGACCAATTTACGCTAAGTTTT3', in reverse orientation into pKB05 after digestion with *Pst*I and *Sal*I. Finally the fused fragments were subcloned into *Pst*I/*Bam*HI-digested pDNeoII [34] to obtain pKB07 (see below, fig. 1).

### Northern blotting

Total RNA was isolated using the RNeasy Mini Kit (Qiagen, Hilden, Germany), chromatographed on 0.9% agarose gels containing 6.6% formaldehyde and blotted onto nylon membranes which were hybridized with DIG-labelled cDNA probes and stained with CSPstar as recommended by the manufacturer (all reagents from Roche Molecular Diagnostics, Mannheim, Germany).

### SDS/polyacrylamide gel electrophoresis and Western blotting

Proteins were chromatographed on 15% polyacrylamide/0.1% SDS gels [35], transferred to nitrocellulose membranes and probed with 1:5000 diluted rabbit antiserum against recombinant *D. discoideum *calcineurin B as described [4].

### Analysis of developmental chimeras

AX2 wild type and mutant strain 01–5 were cultured in AX medium. Cells were washed twice in SP buffer and resuspended at 2 × 10^7 ^cells/ml. The cells were mixed in different ratios and 2 × 10^7 ^cells spread on HABP nitrocellulose filters. Filters were incubated in the dark at 22°C for 30 h.

### Analysis of fruiting body size and spore viability

Wild type and mutant strains were cultured in AX medium. Cells were washed twice in SP buffer and resuspended to 4 × 10^6 ^cells/ml. 100 μl each were spread into individual wells of a 96 well microtiter plate filled with 200 μl SP agar each. Organisms were allowed to develop for 48 h, fruiting bodies were collected, resuspended in SP buffer and frozen at -70°C. After one day spores were spread on nutrient agar with *Klebsiella planticola*, incubated for three days at 22°C and the numbers of clones were counted.

### Embedding of fruiting bodies

*Dictyostelium *fruiting bodies developed on HABP filters were fixed for 3 days in a humid chamber with glutaraldehyde (2.5%) in 0.1 M Na/K phosphate buffer (pH 7.0), using both fixative solution (completely absorbed by the HABP filter) and the arising fixative vapour. The fruiting bodies were then embedded in 1.5% low melting agarose (LGT Agarose, Marine Colloids Inc., US) at 20°C. After hardening the agarose by cooling down to 0°C, blocks including fruiting bodies were prepared. The specimens were then fixed a second time for 2 h at RT with glutaraldehyde in 0.1 M Na/K phosphate buffer (pH 7.0) containing paraformaldehyde (2%) and tannic acid (0.2%). After washing 3 times with the same buffer for 20 min each a third fixation with osmium tetroxyde (1% in 50 mM Na/K phosphate buffer, pH 7.0) for 12 h at 20°C was performed, followed by repeated washing with the same buffer. The specimens were dehydrated in a graded series of ethanol. They were incubated in propylene oxide, followed by a mixture of propylene oxide/epoxide resin (v/v) and pure epoxide resin ERL [36]. The resin was polymerized for 2 days at 60°C.

### Light microscopy

Preparations for light microscopy were made by cutting 1.0 μm sections using a Reichert-Jung Ultracut E ultramicrotome. The sections were fixed onto glass slides by heating for 10 min at 60°C. For histological analysis, sections were stained for 2 min at 60°C with a mixture of Azur II (1% in water) and methylene blue (1% in 1% tetraborate) as described [37], washed in deionised water and dried. Images were obtained with a Leitz Ortholux II microscope using bright field and photographed with a Nikon Digital Camera Cool PIX 990.

### Transmission electron microscopy

Ultrathin sections of ca. 50–80 nm were prepared and mounted on 400 mesh cupper grids. The samples were contrasted with 2% w/v uranyl acetate for 10 min and with 0.2% w/v lead citrate in 0.2 M NaOH for 2 min. Electron micrographs were obtained with a Zeiss EM 10 microscope using Scientia negative films. The negatives were scanned with an Epson 1680 Pro scanner at a resolution of 1200 dpi.

## List of abbreviations used

CN, calcineurin; DIF, differentiation-inducing factor; NCS-1, neuronal calcium sensor-1; NFAT, nuclear factor of activated T cells; GSK, glycogen synthase kinase.

## Authors' contributions

KB carried out most of the experimental work. GT performed the microscopic images. RM participated in the design of the study. BW coordinated the study. All authors read and approved the final manuscript.
